# Predicting protein inter-residue contacts using composite likelihood maximization and deep learning

**DOI:** 10.1186/s12859-019-3051-7

**Published:** 2019-10-29

**Authors:** Haicang Zhang, Qi Zhang, Fusong Ju, Jianwei Zhu, Yujuan Gao, Ziwei Xie, Minghua Deng, Shiwei Sun, Wei-Mou Zheng, Dongbo Bu

**Affiliations:** 1 Key Lab of Intelligent Information Processing, Institute of Computing Technology, Chinese Academy of Sciences, Beijing, China; 2 University of Chinese Academy of Sciences, Beijing, China; 3 Center for Quantitative Biology, School of Mathematical Sciences, Center for Statistical Sciences, Peking University, Beijing, China; 4 Institute of Theoretical Physics, Chinese Academy of Sciences, Beijing, China; 5 College of Life Science and Technology, Huazhong University of Science and Technology, Wuhan, China

**Keywords:** Residue-residue contacts prediction, Deep learning, Markov random fields, Composite likelihood maximization

## Abstract

**Background:**

Accurate prediction of inter-residue contacts of a protein is important to calculating its tertiary structure. Analysis of co-evolutionary events among residues has been proved effective in inferring inter-residue contacts. The Markov random field (MRF) technique, although being widely used for contact prediction, suffers from the following dilemma: the actual likelihood function of MRF is accurate but time-consuming to calculate; in contrast, approximations to the actual likelihood, say pseudo-likelihood, are efficient to calculate but inaccurate. Thus, how to achieve both accuracy and efficiency simultaneously remains a challenge.

**Results:**

In this study, we present such an approach (called clmDCA) for contact prediction. Unlike plmDCA using pseudo-likelihood, i.e., the product of conditional probability of individual residues, our approach uses composite-likelihood, i.e., the product of conditional probability of all residue pairs. Composite likelihood has been theoretically proved as a better approximation to the actual likelihood function than pseudo-likelihood. Meanwhile, composite likelihood is still efficient to maximize, thus ensuring the efficiency of clmDCA. We present comprehensive experiments on popular benchmark datasets, including PSICOV dataset and CASP-11 dataset, to show that: *i*) clmDCA alone outperforms the existing MRF-based approaches in prediction accuracy. *ii*) When equipped with deep learning technique for refinement, the prediction accuracy of clmDCA was further significantly improved, suggesting the suitability of clmDCA for subsequent refinement procedure. We further present a successful application of the predicted contacts to accurately build tertiary structures for proteins in the PSICOV dataset.

**Conclusions:**

Composite likelihood maximization algorithm can efficiently estimate the parameters of Markov Random Fields and can improve the prediction accuracy of protein inter-residue contacts.

**Electronic supplementary material:**

The online version of this article (10.1186/s12859-019-3051-7) contains supplementary material, which is available to authorized users.

## Background

In the natural environment, proteins tend to adopt specific tertiary structural conformations (called *native structures*) that are primarily determined by their amino acid sequences [1]. The native structures are stabilized by local and global interactions among residues, forming inter-residue contacts with proximity [2]. Thus, accurate prediction of inter-residue contacts could provide distance information among residues and thereafter facilitate both free modeling [3–5] and template-based modeling approaches [6] to protein structure prediction.

A great variety of studies have been conducted for predicting inter-residue contacts, which fall into two categories, namely, supervised learning approaches and purely-sequence-based approaches. Supervised learning approaches [7–10] use training sets composed of residue pairs and contact labels indicating whether these residue pairs form contact or not. Machine learning algorithms learn the dependency between contact labels and features of residue pairs, including sequence profile, secondary structure, solvent accessibility. The widely-used machine learning algorithms include neural networks, support vector machines, and linear regression models [11–22]. Recently, Wang et al. applied deep learning techniques to denoise predicted inter-residue contacts, and successfully used predicted contacts to build tertiary structures of several membrane proteins [23].

Unlike the supervised learning approaches, the purely-sequence-based approaches [24–27] do not require any training set that contains known contact labels. Instead, the purely-sequence-based approaches begin with collecting homologous proteins of query protein and constructing multiple-sequence alignment (MSA) of these homologous proteins. Subsequently, coupling columns in MSA are identified to infer contacts among corresponding residues [28, 29]. The underlying principle lies in the fact that protein structures show considerable conservation during evolutionary process; thus, residues in contact tend to co-evolve to maintain the stability of protein structures. Consider two residues being in contact: should one residue mutate and perturb local structural environment surrounding it, its partner would be more likely to mutate into a physicochemically complementary residue to maintain the whole structure. Thus, co-evolving residue pairs, shown as coupling columns in MSA, are high-quality candidates of residues in contacts.

The co-evolution analysis strategy, if considering each residue pair individually, is usually hindered by the entanglement of direct and indirect couplings generated purely by transitive correlations. To disentangle direct couplings from indirect ones, an effective way is to consider all residue pairs simultaneously using a unified model, e.g., Bayesian network [30], Gaussian distribution [21, 31, 32], network deconvolution [33], and Markov random field [34]. Although the Markov random field technique could perfectly model MSA using a joint probability distribution of all residues, the maximization of its actual likelihood function is time-consuming as calculating partition function under multiple parameter settings is needed. To overcome this difficulty, a variety of approximation techniques have been proposed as alternatives to likelihood maximization. For example, bpDCA uses message-passing technique to approximate the actual likelihood [35]; mfDCA employs mean field approximation [26] and successfully uses the predicted contacts in *de novo* protein structure prediction, and plmDCA completely avoids the calculation of partition function by using pseudo-likelihood as approximation to the actual likelihood and outperforms mfDCA in prediction accuracy [36, 37].

There is a dilemma in MRF-based approaches to contact prediction: the actual likelihood function of MRF model is accurate but time-consuming to calculate; in contrast, its approximations, say pseudo-likelihood used by plmDCA, are usually efficient to calculate but inaccurate. Thus, how to achieve both accuracy and efficiency simultaneously remains a challenge to the prediction of inter-residue contacts.

In this study, we present such an approach that achieves both accuracy and efficiency simultaneously. Unlike plmDCA applying pseudo-likelihood to approximate the actual likelihood function, our approach applied composite likelihood maximization for direct coupling analysis and was therefore named as clmDCA. Pseudo-likelihood uses the product of conditional probability of individual residues whereas composite likelihood uses the product of conditional probability of all residue pairs and thus is more consistent with the objective of predicting inter-residue contacts. On one side, the composite likelihood has been theoretically proved as a better approximation to the actual likelihood function than pseudo-likelihood. On the other side, composite likelihood is still efficient to maximize, which ensures the efficiency of clmDCA. We also investigated the compatibility of clmDCA with subsequent refinement procedure using the deep neural network technique.

We present comprehensive experiments on popular benchmark datasets, including PSICOV dataset and CASP-11 dataset. Experimental results suggested that: *i*) clmDCA alone outperforms the existing purely-sequence-based approaches in prediction accuracy. *ii*) When enhanced with deep learning technique for denoising, the prediction accuracy of clmDCA was further significantly improved. Compared with plmDCA, clmDCA is more suitable for subsequent refinement by deep learning. We further successfully applied the predicted contacts to accurately build structures of proteins in the PSICOV dataset.

## Results

### Test datasets and Evaluation measure

In our experiments, we tested clmDCA on PSICOV [21] dataset and CASP11 dataset. PSICOV dataset contains 150 proteins and each protein has a highly resolved (resolution ≤1.9Å) X-ray crystallographic structure available and the length ranges from 50 to 275; CASP11 dataset is from CASP11 experiments and contains 85 proteins[38]. We built the MSAs using HHblits with options “-n 3 -e 0.001 -id 90 -cov 70" and with sequence database uniprot20_2015_06.

To train the deep residual network for refinement, we constructed a training datasets by selecting a subset (protein sequence length <350 AA) from the training datasets used in Ref. [39]. To avoid possible overlap between training datasets and testing datasets, we filtered out the similar proteins shared by training datasets and test datasets. The criterion of similarity was set as sequence identity over 25%, which has been widely used in previous studies [9, 32, 40]. BLAST was used to generate the pairwise alignments when we calculated the sequence identity [41]. After this filtering operation, the training set contains 3705 proteins in total (available through http://protein.ict.ac.cn/clmDCA/ContactsDeepTraining.tar). 500 proteins were randomly selected as validation dataset.

We measured the number of non-redundant sequence homologs in MSA by *N*_*eff*_ as follows [36].

$$N_{eff} = \sum_{i} \frac{1} {|\{ j | S_{ij} < 80\%\}|} $$

where both *i* and *j* go over all the sequence homologs, and *S*_*ij*_ is a binary similarity value between two proteins.

For each protein in training and test datasets, true contacts have been annotated between two residues with a *C*_*β*_−*C*_*β*_ (*C*_*α*_ in the case of Glycine residues) distance of less than 8Å. The performance of contact prediction was evaluated using the mean prediction precision (also known as accuracy), i.e., the fraction of predicted contacts are true [21, 26, 32, 37, 40].

### Overall performance on pSICOV and cASP-11 datasets

Table 1 summarizes the performance of clmDCA, plmDCA, PSICOV and mfDCA on the PSICOV dataset. Following the contact prediction conventions, we filtered out short distance contacts under two settings of sequence separation thresholds (6 AA and 23 AA), and reported the accuracies of top *L*/10,*L*/5,*L*/2, and *L* predicted contacts.

**Table 1:** Contact prediction accuracy on PSICOV benchmark

Methods	*separation*≥6				*separation*>23			
	*L*/10	*L*/5	*L*/2	*L*	*L*/10	*L*/5	*L*/2	*L*
PSICOV	0.77	0.72	0.58	0.44	0.72	0.64	0.47	0.34
mfDCA	0.73	0.67	0.57	0.44	0.71	0.64	0.49	0.36
plmDCA	0.81	0.77	0.66	0.51	0.78	0.71	0.56	0.40
clmDCA	0.83	0.80	0.70	0.55	0.81	0.75	0.61	0.45
plmDCA+DL	0.92	0.90	0.85	0.75	0.89	0.86	0.74	0.59
clmDCA+DL	0.94	0.92	0.86	0.77	0.91	0.86	0.76	0.61

**Fig. 1:**
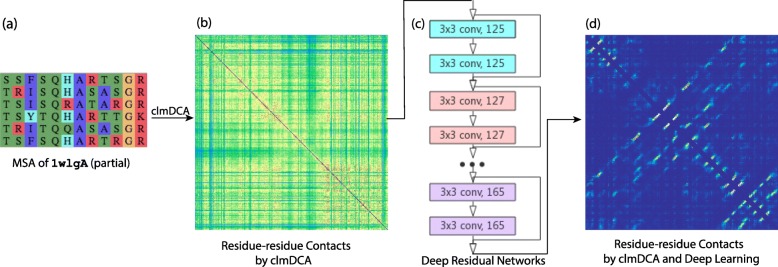
Predicted contacts (top *L*/5; sequence separation >6 AA) for protein structure with PDB ID: 1ne2A by plmDCA and clmDCA. Red (green) dots indicate correct (incorrect) prediction, while grey dots indicate all true residue-residue contacts. **a** The comparison between clmDCA (in upper-left triangle) and plmDCA (in lower-right triangle). **b** The comparison between clmDCA (in upper-left triangle) and clmDCA after refining using deep residual network (in lower-right triangle)

As shown in Table 1 and Fig. 1, clmDCA outperforms plmDCA and other purely-sequence-based approaches. Take top *L*/10 predictions with the sequence separation threshold 6*AA* as an example. clmDCA achieved prediction precision of 0.83, which is higher than plmDCA (0.81), mfDCA (0.73) and PSICOV (0.77).

Table 2 shows that on the CASP-11 dataset, the prediction accuracies of all these approaches are relatively lower than those on the PSICOV dataset. This might be attributed to the difference in MSA quality: the median number of non-redundant homologous proteins is 2374 for proteins in PSICOV dataset, which is substantially higher than that in CASP-11 dataset (352 homologous proteins on average); the analysis of the effect of the number of effective homologous proteins is shown in the following section. Table 2 suggested that even if the MSA quality is low, clmDCA still outperformed other approaches.

**Table 2:** Contact prediction accuracy on CASP-11 targets

Methods	*separation*≥6				*separation*>23			
	*L*/10	*L*/5	*L*/2	*L*	*L*/10	*L*/5	*L*/2	*L*
PSICOV	0.54	0.48	0.39	0.31	0.49	0.43	0.33	0.24
mfDCA	0.49	0.44	0.37	0.30	0.48	0.42	0.33	0.25
plmDCA	0.54	0.49	0.41	0.33	0.51	0.45	0.36	0.26
clmDCA	0.57	0.53	0.44	0.36	0.53	0.49	0.38	0.29
plmDCA + DL	0.77	0.71	0.60	0.48	0.50	0.46	0.38	0.29
clmDCA + DL	0.86	0.81	0.72	0.60	0.69	0.64	0.52	0.40

These tables also suggest that when equipped with deep learning techniques for refinement, both plmDCA and clmDCA achieved better prediction accuracy. For example, on the CASP-11 dataset, plmDCA and clmDCA alone achieved prediction accuracy of only 0.54 and 0.57, respectively (sequence separation >6*AA*; top *L*/10 contacts). In contrast, by applying the deep learning technique for refinement, the prediction accuracies significantly increased to 0.77 and 0.86, respectively. More importantly, the improvement of clmDCA (from 0.57 to 0.86) is considerably higher than that of plmDCA (from 0.54 to 0.77), suggesting that clmDCA results are more suitable for refinement using deep learning technique.

### Comparison of plmDCA and clmDCA: a case study

**Fig. 2:**
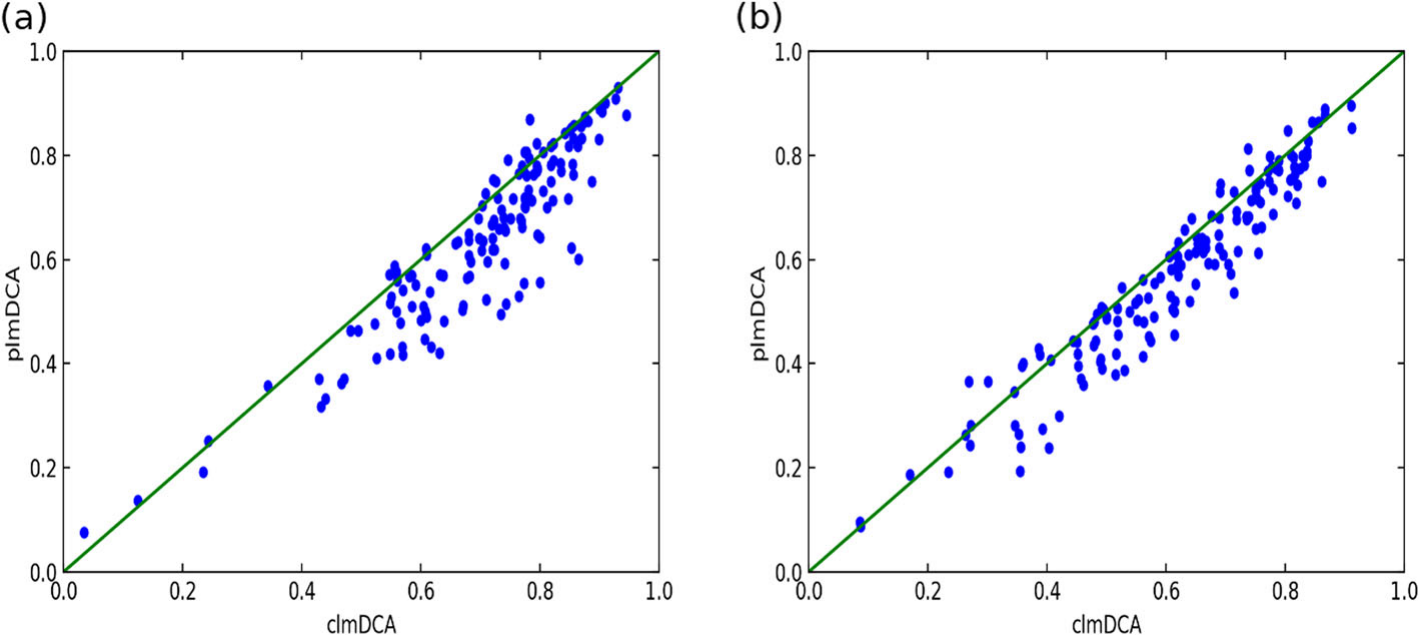
The relationship between the prediction accuracy and quality of MSA. Here the quality of MSA is measured using *N*_*eff*_, i.e. the number of effective homologous sequences. Dataset: PSICOV. Sequence separation: >6 AA

In Fig. 2, we present the predicted contacts for protein structure with PDB ID: 1ne2A by using plmDCA and clmDCA. By comparing with true contacts, we observed that clmDCA achieved a contact prediction precision of 0.92, which is significantly higher than plmDCA (prediction precision: 0.50).

The two approaches, plmDCA and clmDCA, differ only in the way to calculate the parameters *h*_*i*_ and *e*_*ij*_ and thereafter the coupling strength *J*_*ij*_. To reveal this difference, we examined two residue pairs, one being in contact, and the other non-contact. As shown in Additional file 1: Figure S1 (a), the non-contact residue pair ALA183-ILE189 was incorrectly reported as being in contact by plmDCA (coupling strength: *J*_183,189_=1.63; rank: 14th). In comparison, this pair was ranked 2053th by clmDCA (coupling strength: *J*_183,189_=0.05) and was not reported as being in contact.

Additional file 1: Figure S1 (b) shows THR75-MSE97 as an example of contacting residue pair. This pair was ranked 40th by plmDCA due to its considerably small coupling strength *J*_75,97_=1.34. On the contrary, clmDCA calculated the coupling strength as 0.58 (rank: 12th) and thus correctly reported it as a contact. Together these results suggest that compared with plmDCA, clmDCA assigned higher ranks for true contacts.

### Examining the factors affecting contact prediction

The purely-sequence-based approaches use MSA as sole information source; thus, their performances are largely affected by the quality of MSA that is commonly measured using *N*_*eff*_. Most purely-sequence-based approaches perform perfectly for query protein with high quality, say *N*_*eff*_≥1000; thus, it is important for a prediction approach to work perfectly when high-quality MSAs are unavailable [10, 21, 42].

**Fig. 3:**
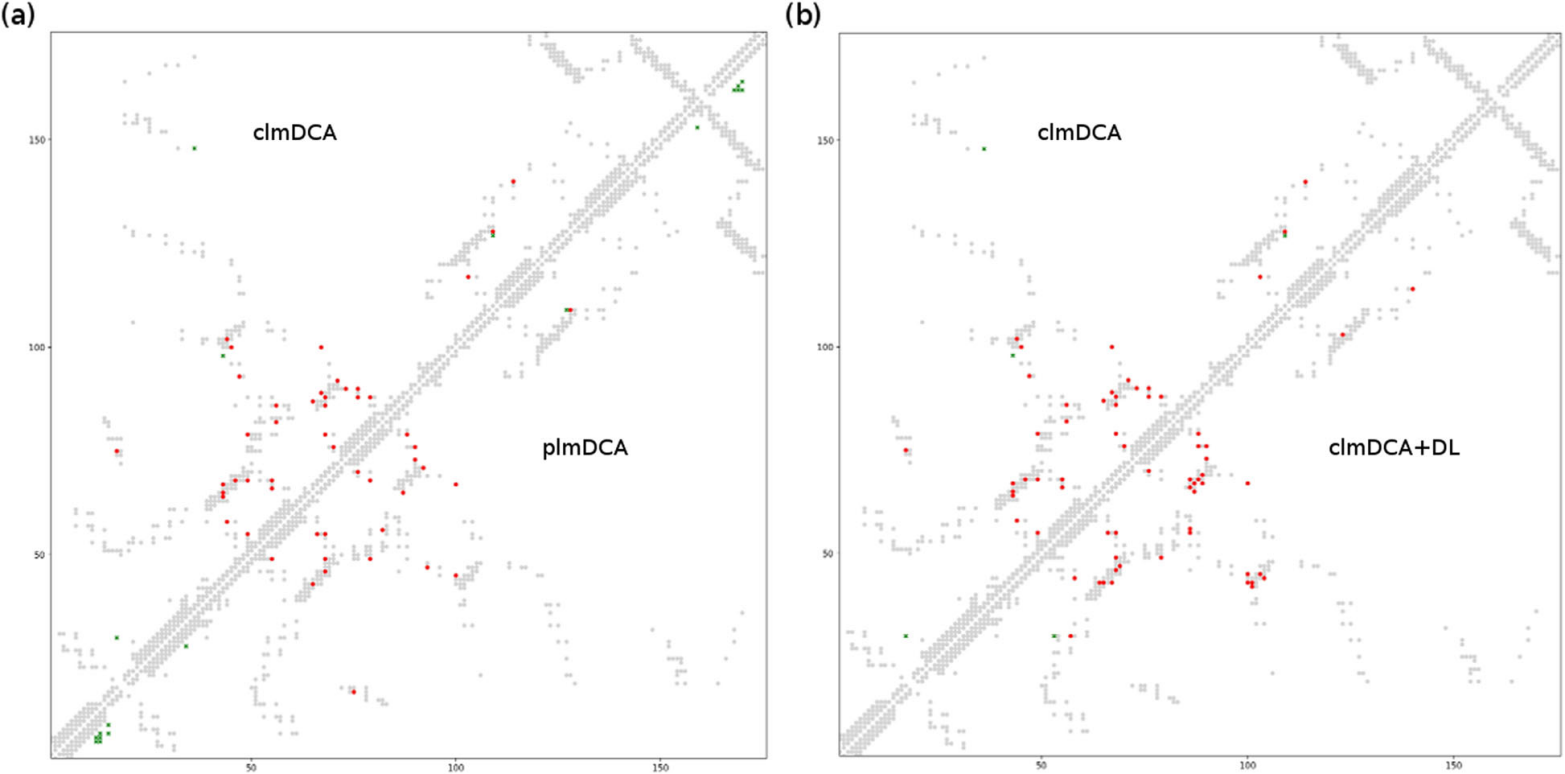
Native structure and predicted structures for protein structure with PDB ID: 1vmbA. **a** Native structure. **b** Structure built using contacts predicted by plmDCA (TMscore: 0.42). **c** Structure built using contacts predicted by clmDCA alone (TMscore: 0.55). **d** Structure built using contacts predicted by clmDCA together with deep learning for refinement (TMscore: 0.72)

Here we examined the effects of *N*_*eff*_ on the prediction accuracy of clmDCA. To this end, we divided the proteins in the PSICOV dataset into four groups according to *N*_*eff*_ of their MSAs, and calculated the prediction accuracy for each group individually. As shown in Fig. 3, the prediction accuracy of plmDCA, mfDCA, clmDCA and PSICOV increases with *N*_*eff*_ as expected. Remarkably, clmDCA outperforms all other approaches even if *N*_*eff*_ is only 523, which clearly shows the robustness of clmDCA.

### Building protein 3D structures using the predicted inter-residue contacts

We further applied the predicted inter-residue contacts to build 3D structures of the query proteins. For this aim, we run CONFOLD [43] with predicted contacts as input. CONFOLD builds a protein structure that satisfies the input inter-residue contacts as well as possible. Previous studies have shown that knowing only a few true contacts is sufficient for building high-quality 3D structures [44].

Additional file 1: Figure S2 compares the quality of structures built using top *L* contacts predicted by plmDCA, clmDCA alone, and clmDCA together with deep learning. When using contacts predicted by clmDCA alone, the quality of built structures are the same to those built using contacts by plmDCA; however, the combination of clmDCA and deep learning techniques showed substantial advantage. Specifically, when using top *L* contacts predicted by plmDCA as input, we successfully built high-quality structures for 77 proteins in the PSICOV dataset (TMscore >0.6). In contrast, we built high-quality structures for 78 proteins when using predicted contacts by clmDCA. By enhancing clmDCA with deep learning techniques, the number of high-quality predictions further increased to 80.

**Fig. 4:**
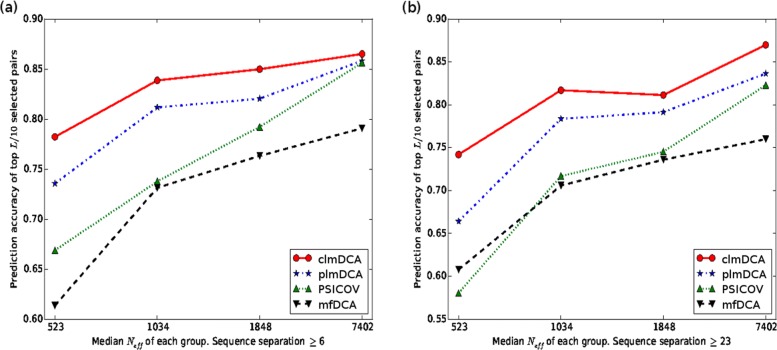
Procedure of clmDCA to predict inter-residue contacts. **a** For a query protein (1wlg_A as an example), we identified its homologues by running HHblits [59] against *nr90* sequence database (parameter setting: *j*:3,*id*:90,*cov*:70) and constructed multiple sequence alignment of these proteins. **b** The correlation among residues in MSA was disentangled using composite likelihood maximization technique, generating prediction of inter-residue contacts. **c** The predicted contacts were fed into a deep neural network for refinement. **d** The refined prediction of inter-residue contacts

A concrete example is shown in Fig. 4: For protein structure with PDB ID: 1vmbA, the predicted structure has just medium quality (TMscore: 0.55) when using predicted contacts by clmDCA alone. In contrast, when using refined contacts, the quality of predicted protein structure increased to 0.72. These results demonstrate the effectivity of clmDCA, especially when equipped with deep learning techniques, in predicting 3D structures.

## Discussion

In this study, we present an approach to predict inter-residue contacts based on composite-likelihood maximization. Like pseudo-likelihood, composite likelihood is also an approximationx to the actual likelihood of Markov random field model and thus avoids the inefficiency in calculating partition function. Compared with pseudo-likelihood, composite likelihood is much closer to the true likelihood and is more suitable for the subsequent refinement procedure based on deep learning. We present comprehensive results to show that the composite-likelihood technique outperforms the existing approaches in terms of prediction accuracy. The predicted contacts were also proved to be useful to predict high-quality structures of query proteins. Together, these results suggest that composite likelihood could achieve both prediction accuracy and efficiency simultaneously.

We also have tried a hybrid likelihood that combines pseudo-likelihood and the composite likelihood. Experimental results (data not shown here) suggested that this hybrid likelihood achieved prediction accuracy comparable to the application of composite likelihood alone, implying that the correlation information extracted by pseudo-likelihood is nearly completely contained within that extracted by the composite likelihood.

The composite likelihood used in this study is pairwise or 2-order, i.e., we consider the conditional probability of all possible residue pairs. A natural extension is 3- or higher-order composite likelihood that considers the conditional probability of all possible 3 or more residue combinations. Compared with the pairwise composite likelihood, the 3-order composite likelihood showed merely marginal improvement on prediction accuracy but significantly lower efficiency (see Additional file 1: Table S2). Thus it is not necessary to apply the 3- or higher-order composite likelihood technique in practice.

In this study, we applied the gradient descent technique to maximize the composite likelihood. An alternative technique is Gibbs sampling or contrastive divergence, which has been shown in training restricted Boltzmann machine [45, 46]. In addition, a generalization of pairwise composite likelihood is tree-reweighted belief propagation [47]. To further speed up clmDCA, a reasonable strategy is to model residue pairs reported by plmDCA only rather than all possible residue pairs. The implementation of these techniques will be our future work of this research.

## Conclusions

In conclusion, clmDCA can efficiently estimate the parameters of Markov Random Fields and can improve the prediction accuracy of protein inter-residue contacts. In addition, the prediction accuracy of clmDCA was further significantly improved by deep learning methods.

## Methods

### Framework of our methods

**Fig. 5:**
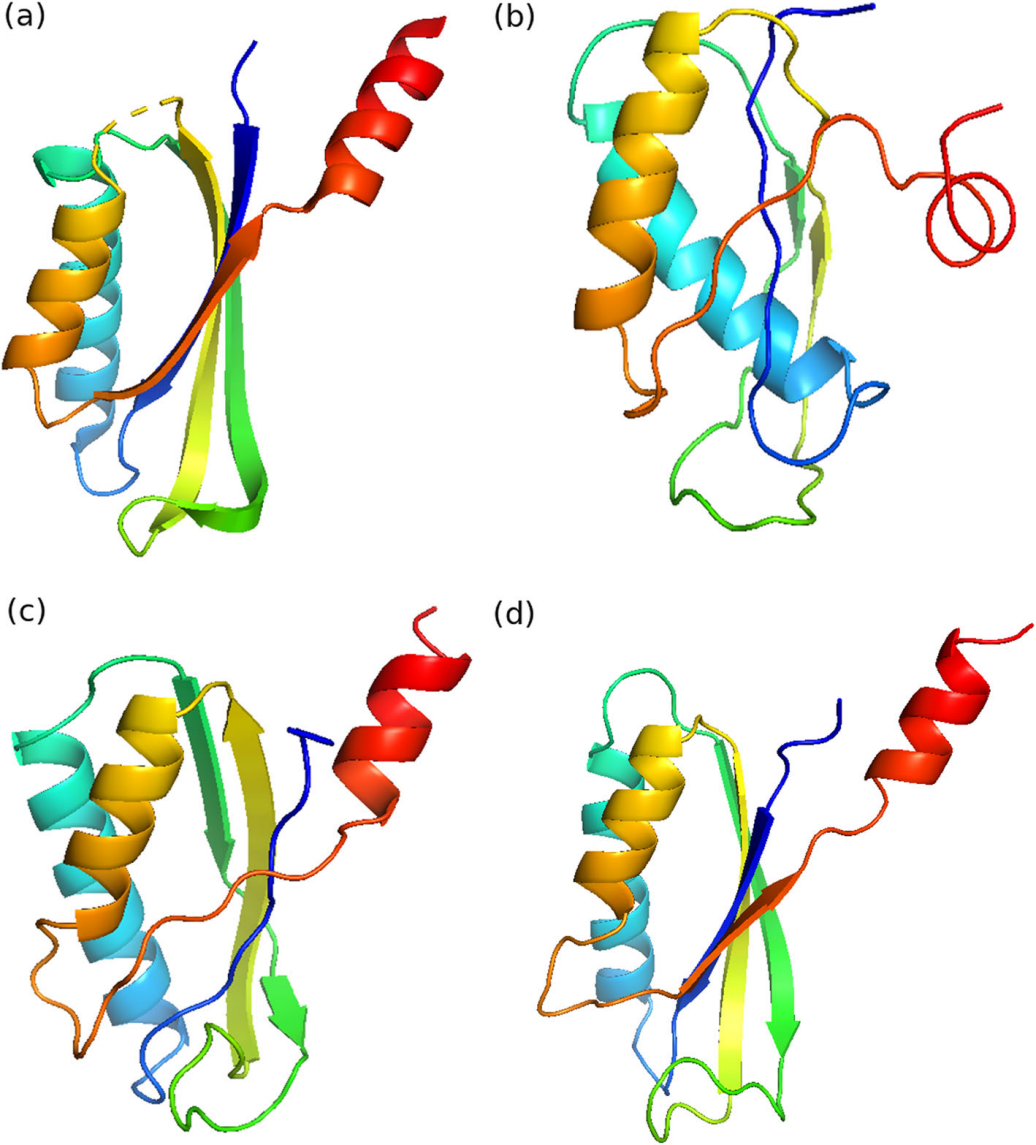
Comparison of prediction accuracy of top *L*/2 contacts reported by plmDCA(*y*-axis) and clmDCA(*x*-axis) with two sequence separation threshold on the PSICOV dataset. **a** Sequence separation >6 AA. **b** Sequence separation >23 AA

For a query protein, clmDCA predicts its inter-residue contacts through the following three steps (Fig. 5). First, we construct multiple sequence alignment (MSA) for homologous proteins of the query protein. According to the MSA, the correlations among residues are disentangled using the composite likelihood maximization technique, and are subsequently explored to infer contacts among residues. The generated inter-residue contacts are further refined using a deep residual network.

### Modeling mSA using markov random field

For a query protein of length *L*, we denote an MSA of its homologous proteins as $\{x^{m}\}_{m=1}^{M}$, where *M* denotes the number of homologous proteins, and $x^{m}=(x^{m}_{1}, x^{m}_{2},..., x^{m}_{L})$ represents the *m*-th protein sequence in the MSA. Each element $x^{m}_{i}, i=1,2,..., L$, has a total of 21 possible values, representing 20 ordinary amino acid types and gap in alignment (For the sake of simplicity, we treat gap as a special amino acid type).

We use a vector of variables *X*=(*X*_1_,*X*_2_,⋯,*X*_*L*_) to represent a protein sequence in MSA with *X*_*i*_ representing position *i* of MSA. According to the maximum entropy principle [34], the probability that *X* takes a specific value *x*^*m*^ can be represented using Markov random field model [26]:

1 $$ {\begin{aligned} P(X=x^{m}) = \frac{1}{Z^{m}} \exp\left\{\sum_{i=1}^{L}h_{i}(x_{i}^{m}) + \sum_{i=1}^{L}\sum_{j=i+1}^{L} e_{ij}\left(x_{i}^{m}, x_{j}^{m}\right)\right\} \end{aligned}}  $$

Here the singleton term *h*_*i*_(*a*) encodes the propensity for amino acid type *a* to appear at position *i*, whereas the doubleton term *e*_*i*,*j*_(*a*,*b*) encodes the coupling strength between position *i* and *j* when amino acid types *a* and *b* appear at these positions, respectively. *Z*^*m*^ denotes a partition function acting as a global normalizer to ensure the probabilities of all possible values of *X* sum to 1.

The optimal parameters *h*_*i*_(*a*) and *e*_*i*,*j*_(*a*,*b*) can be solved via maximizing the likelihood (in logarithm) of all homologous proteins in the MSA, i.e.,

2 $$ \mathcal{L} = \frac{1}{M} \sum_{m=1}^{M}\log P(X=x^{m})  $$

Finally, we calculated the coupling strength between position *i* and *j* using Frobenius form [21] of the matrix *e*_*ij*_:

3 $$ J_{ij} = \left(\sum_{a=1}^{21}\sum_{b=1}^{21} e_{ij}^{2}(a, b) \right)^{\tfrac{1}{2}},  $$

which was used to measure the possibility for the corresponding residues of the query protein being in contact.

### Direct coupling analysis using composite likelihood maximization

The maximization of the actual likelihood of MRF model is inefficient since the calculation of partition function *Z*^*m*^ under multiple parameter settings is needed [26, 35]. To circumvent this difficulty, pseudo-likelihood was used as an approximation to the actual likelihood $\mathcal {L}$ [36, 37]:

4 $$ \mathcal{P}\mathcal{L} = \frac{1}{M} \sum_{m=1}^{M} \sum_{i=1}^{L} \log P\left(X_{i}=x^{m}_{i} | X_{\neg i}=x^{m}_{\neg i}\right)  $$

Here $P\left (X_{i}=x^{m}_{i} | X_{\neg i}=x^{m}_{\neg i}\right)$ represents the conditional probability for amino acid type $x^{m}_{i}$ appearing at position *i* given the other positions’ value $x^{m}_{\neg i}$. Unlike the actual likelihood $\mathcal {L}$, the approximation $\mathcal {P}\mathcal {L}$ is easy to maximize; however, the deviation between $\mathcal {L}$ and $\mathcal {P}\mathcal {L}$ is large, causing inaccurate estimation of parameters in *e*_*ij*_ and thereafter inaccurate prediction of inter-residue contacts.

To better approximate the actual likelihood $\mathcal {L}$, we use composite likelihood $\mathcal {C}\mathcal {L}$ instead of pseudo-likelihood $\mathcal {P}\mathcal {L}$ [48]. The composite likelihood is defined as:

5 $$ \mathcal{C}\mathcal{L} = \frac{1}{M}\sum_{m=1}^{M} \sum_{c\in C} \log P\left(X_{c} = x_{c}^{m} | X_{\neg {c}} = x_{\neg {c}}^{m}\right)  $$

Here *C* denotes subsets of variables. This way, the correlations among all variables within each subset in *C* are taken into account by $\mathcal {C}\mathcal {L}$.

It should be pointed out that composite likelihood is a general model with $\mathcal {L}$ and $\mathcal {P}\mathcal {L}$ as its special cases. In particular, when setting *C*={{1,2,⋯,*L*}}, composite likelihood $\mathcal {C}\mathcal {L}$ degenerates to the actual likelihood $\mathcal {L}$. On the contrary, when setting *C*={{1},{2},⋯,{*L*}}, the composite likelihood $\mathcal {C}\mathcal {L}$ reduces into the pseudo-likelihood $\mathcal {P}\mathcal {L}$.

To match our objective of predicting inter-residue contacts, we set *C* as all possible residue pairs, i.e., *C*={{1,2},{1,3},⋯,{*i*,*j*},⋯,{*L*−1,*L*}}. This way, the actual likelihood is approximated using pairwise composite likelihood, which explicitly represents conditional probabilities of all residue pairs as below.

6 $$ {\begin{aligned} \mathcal{C}\mathcal{L}_{\text{pairwise}} &=\frac{1}{M}\sum_{m=1}^{M}\sum_{i=1}^{L}\sum_{j>i}^{L}\log P\left(X_{i,j}=x_{i,j}^{m}|X_{\neg\{i,j\}}=x_{\neg\{i,j\}}^{m}\right)\\ &=\frac{1}{M}\sum_{m=1}^{M}\sum_{i=1}^{L}\sum_{j>i}^{L}\log \frac{1}{Z_{ij}^{m}}\exp\left\{ h_{i}\left(x_{i}^{m}\right) + h_{j}\left(x_{j}^{m}\right) +e_{ij}\left(x_{i}^{m}, x_{j}^{m}\right)\right. \\ &\left.+ \sum_{k\neq i, k \neq j}\left[e_{ik}(x_{i}^{m}, x_{k}^{m}) + e_{jk}(x_{j}^{m}, x_{k}^{m})\right]\right\} \end{aligned}}  $$

in which *Z*_*ij*_ is a partition function. To find optimal parameters *h*_*i*_ and *e*_*ij*_ such that $\mathcal {C}\mathcal {L}_{\text {pairwise}}$ is maximized, we employed the classical Broyden-Fletcher-Goldfarb-Shanno algorithm with efficient calculation of gradients (See Additional file 1 for details).

The advantages of pairwise composite likelihood technique are two-folds: *i*) Compared with pseudo-likelihood, pairwise composite likelihood is a better approximation to the actual likelihood. To be more precise, it has been proven that under any specific parameter setting, $\mathcal {P}\mathcal {L} \leq \mathcal {C}\mathcal {L}_{\text {pairwise}} \leq \mathcal {L}$ [49]. *ii*) The gradients of $\mathcal {C}\mathcal {L}_{\text {pairwise}}$ can be calculated in polynomial time. Thus, the pairwise composite likelihood approach achieves both accuracy and efficiency simultaneously.

### Refining inter-residue contacts using deep residual network

The MRF-based approaches, even being enhanced with direct coupling analysis technique, usually show limited prediction accuracy as they explore MSA of the query protein only but never considers known contacts of other proteins for reference. Recent progress suggested that this limitation could be effectively avoided by integrating MRF-based approaches with supervised learning approaches, especially deep neural networks [39, 42, 50, 51]. The power of this integration strategy is rooted in the complementary properties between these two types of approaches: *i*) The MRF technique considers inter-residue contacts individually but never consider the interdependency among contacts, say *clustering pattern* of contacts existing in *β* sheets. *ii*) In contrast, deep neural networks could learn such contact patterns from known contacts of proteins in training sets, which could be exploited to identify and therefore filter out erroneous predictions by MRF-based approaches.

#### Input features

We also included other features besides plmDCA scores. In particular, the input features include protein sequence profile, predicted secondary structure and solvent accessibility. Here, protein sequence profile was calculated using HHblits[52], and secondary structure and solvent accessibility were predicted using RaptorX-Property[53]. For a pair of residues *i* and *j*, we concatenate the features of residue *i* and residue *j* to a single vector and combine it with plmDCA score as one input feature of this residue pair.

#### Model architecture

We used deep residual networks [39, 54] to integrate clmDCA score and other input features. Skipping layers in deep residual network can speed up training by reducing the impact of vanishing gradient and hence make it possible to train ultra-deep networks effectively. Here, we used a total of 42 convolution layers, organized into 21 residual blocks (Fig. 4.c). For each convolutional layer, we set the kernel size as 3×3 and use *ReLU* as our activation function [55]. The final layer is *softmax* that transforms the final predicted possibility into the range [0,1].

#### Loss function

We used cross entropy loss as our loss function. Besides, we added *L*_2_−norm regularization to our loss function to ease over-training issue. We set regularization factor as 1*e*−4.

#### Model training

We used Adam algorithm to minimize the objective function with hyperparameters *lr*=1*e*−4,*β*=0.99 and *ε*=1*e*−8[56]. 500 out of 3275 training protein structures are randomly selected as the validation dataset. And early stopping was performed during training. The whole algorithm is implemented by TensorFlow and mainly runs on GPU.

No fully-connected layers were used which makes our architecture as fully convolutional networks. Hence, our network can deal with proteins with different lengths. In particular, we applied zero padding for each minibatch so that each training sample has the same length with the longest one in its minibatch. We also filtered out the padded positions when we aggregated the final training loss. We set our training batch size as 2. We did not try a larger batch size due to the limit of our GPU memory.

### Programs to compare

To evaluate prediction accuracy, we compared our method clmDCA with several popular methods including plmDCA[36, 57], mfDCA [26] and PSICOV[21]. We run these programs with their default options on the same MSAs built by HHblits.

To fairly compare the performance of plmDCA+DL and clmDCA+DL, deep learning models with the same architecture were trained separately for plmDCA and clmDCA.

## Additional file

Additional file 1 The additional results on the performance of clmDCA. Figure S1 shows a case study of clmDCA. Figure S2 shows the comparison of the quality of the structures built using predicted contacts. Figure S3 shows the run time of clmDCA. Table S1 shows the time complexity for calculating likelihood function. Table S2 shows the performance of 3-order clmDCA. (PDF 721 kb)

## Data Availability

The software clmDCA and a server are publicly accessible through http://protein.ict.ac.cn/clmDCA/. The datasets used in the current study are available from the corresponding author on a reasonable request.

## Abbreviations

MRF: Markov random field
MSA: Multiple sequence alignment
