# Correction to: Predicting protein inter-residue contacts using composite likelihood maximization and deep learning

**DOI:** 10.1186/s12859-019-3198-2

**Published:** 2019-11-29

**Authors:** Haicang Zhang, Qi Zhang, Fusong Ju, Jianwei Zhu, Yujuan Gao, Ziwei Xie, Minghua Deng, Shiwei Sun, Wei-Mou Zheng, Dongbo Bu

**Affiliations:** 1 Key Lab of Intelligent Information Processing, Institute of Computing Technology, Chinese Academy of Sciences, Beijing, China; 2 University of Chinese Academy of Sciences, Beijing, China; 3 Center for Quantitative Biology,School of Mathematical Sciences, Center for Statistical Sciences, Peking University, Beijing, China; 4 Institute of Theoretical Physics, Chinese Academy of Sciences, Beijing, China; 5 College of Life Science and Technology, Huazhong University of Science and Technology, Wuhan, China

**Correction to: BMC Bioinformatics (2019) 20:537**

**https://doi.org/10.1186/s12859-019-3051-7**

Following publication of the original article [1], the author explained that there are several errors in the original article;

1. The figures’ order in HTML and PDF does not match with each other.

2. The figures are incorrect order; the images do not match with the captions.

In this correction article the figures are shown correct with the correct captions.

**Fig. 1:**
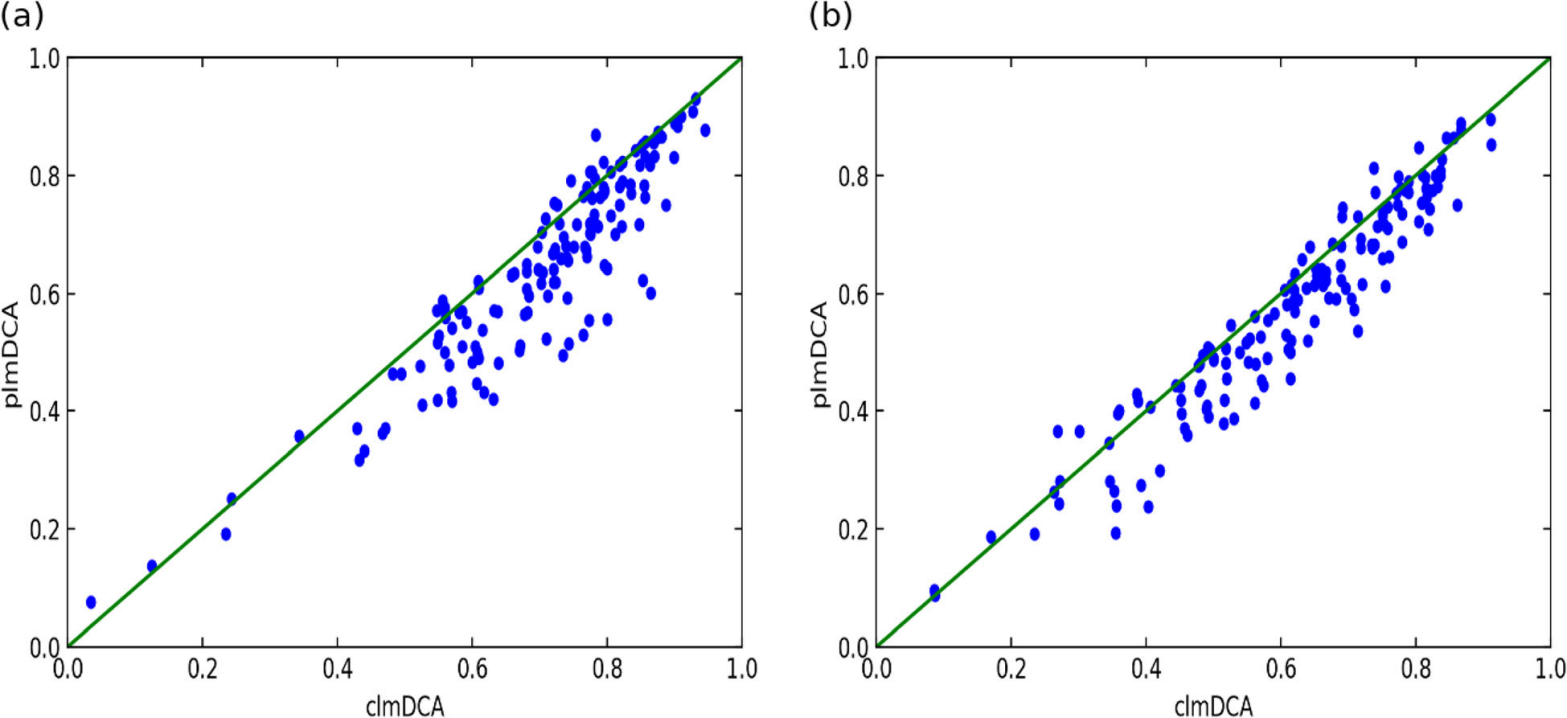
Comparison of prediction accuracy of top L/2 contacts reported by plmDCA(y-axis) and clmDCA(x-axis) with two sequence separation threshold on the PSICOV dataset. **a** Sequence separation >6 AA. **b** Sequence separation >23 AA

**Fig. 2:**
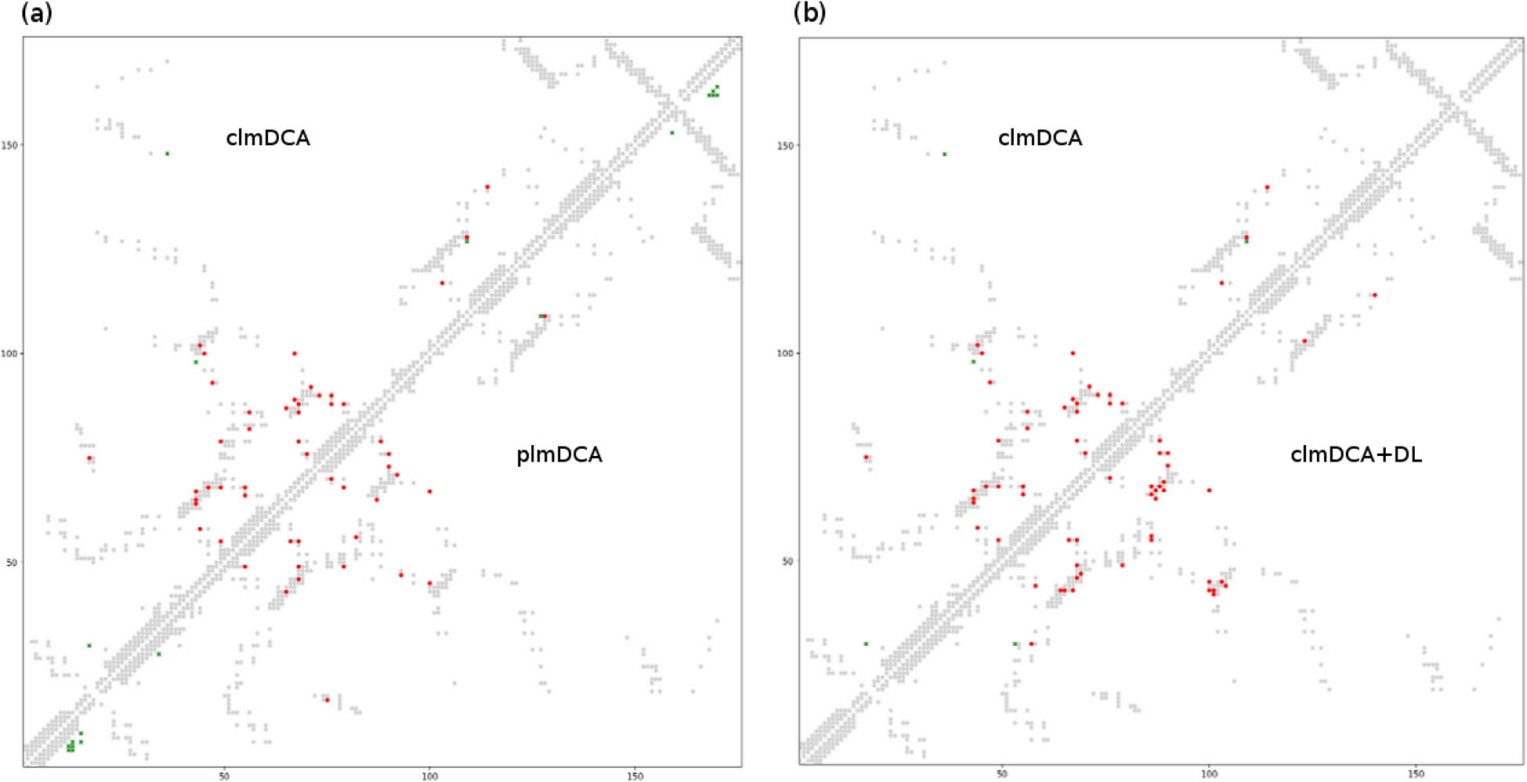
Predicted contacts (top L/5; sequence separation >6 AA) for protein structure with PDB ID: 1ne2A by plmDCA and clmDCA. Red (green) dots indicate correct (incorrect) prediction, while grey dots indicate all true residue-residue contacts. **a** The comparison between clmDCA (in upper-left triangle) and plmDCA (in lower-right triangle). **b** The comparison between clmDCA (in upper-left triangle) and clmDCA after refining using deep residual network (in lower-right triangle)

**Fig. 3:**
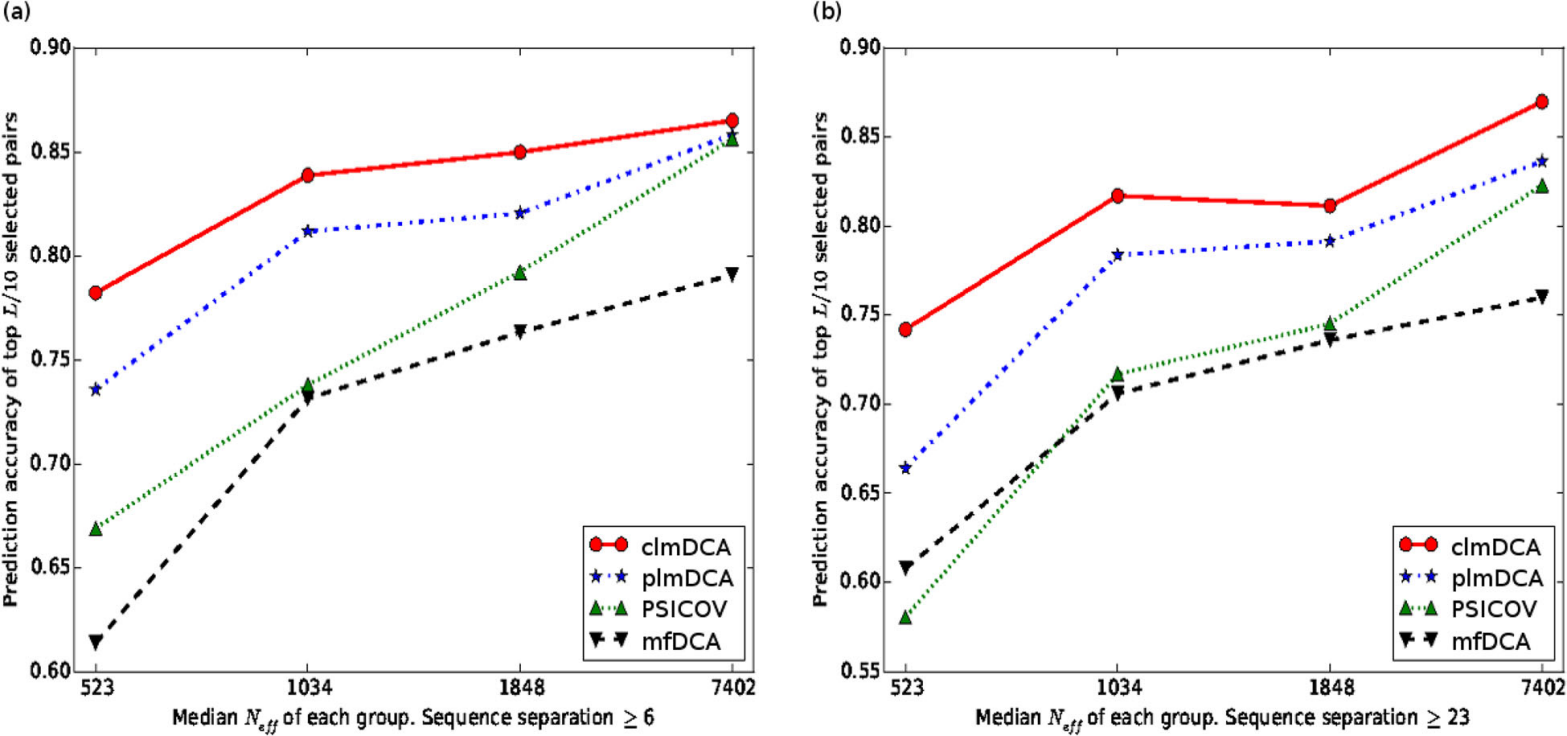
The relationship between the prediction accuracy and quality of MSA. Here the quality of MSA is measured using Neff, i.e. the number of effective homologous sequences. Dataset: PSICOV. Sequence separation: > 6 AA

**Fig. 4:**
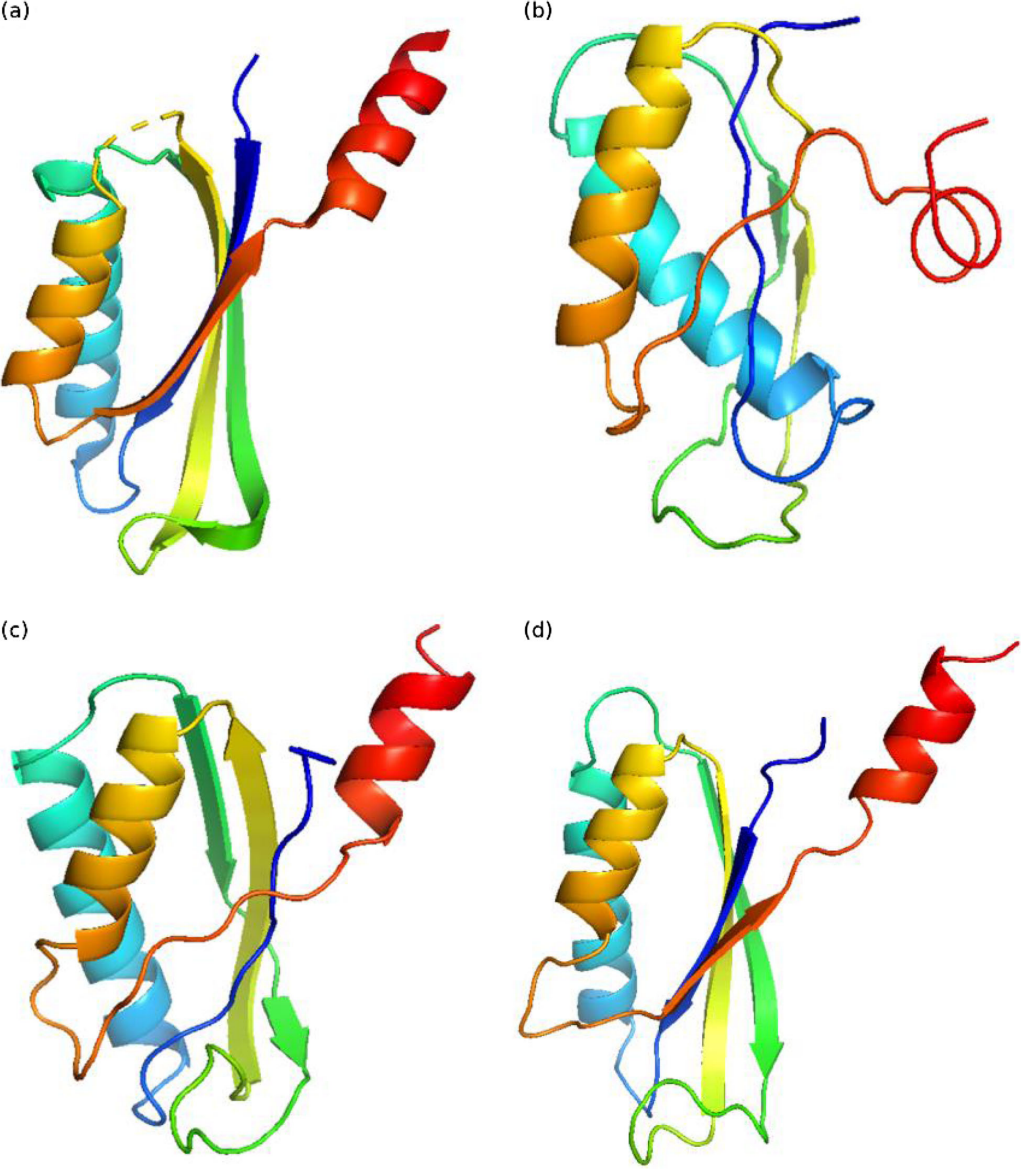
Native structure and predicted structures for protein structure with PDB ID: 1vmbA. **a** Native structure. **b** Structure built using contacts predicted by plmDCA (TMscore: 0.42). **c** Structure built using contacts predicted by clmDCA alone (TMscore: 0.55). **d** Structure built using contacts predicted by clmDCA together with deep learning for refinement (TMscore: 0.72)

**Fig. 5:**
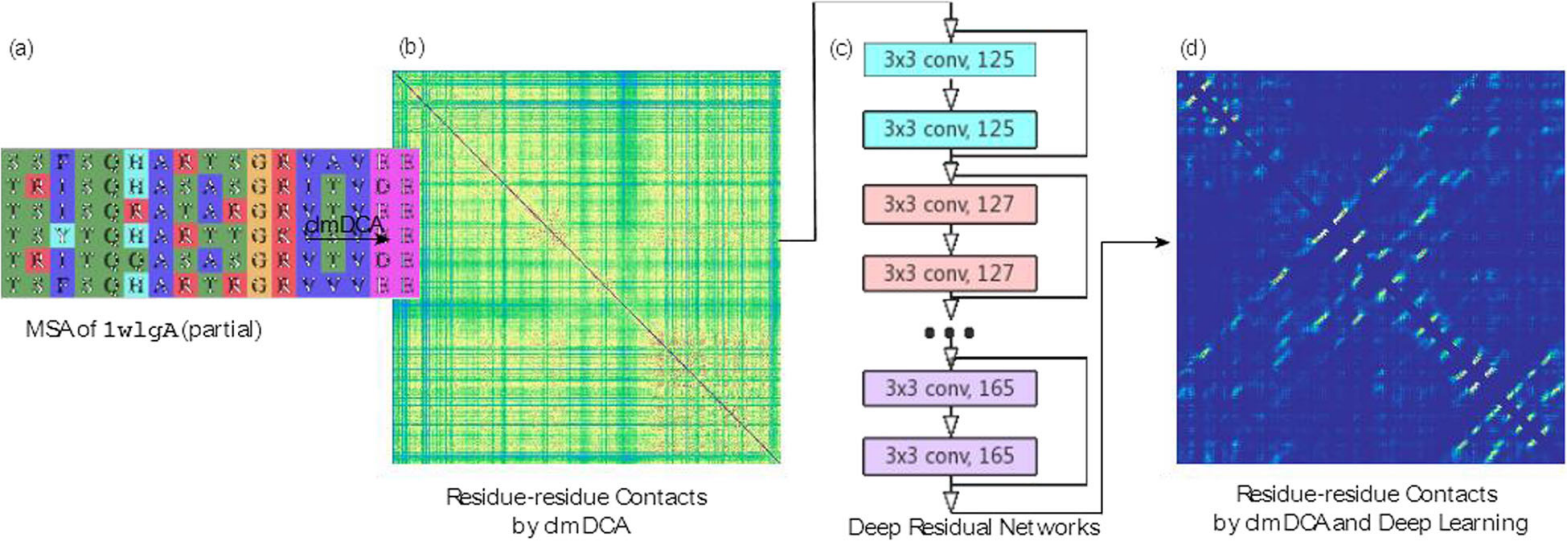
Procedure of clmDCA to predict inter-residue contacts. **a** For a query protein (1wlg_A as an example), we identified its homologues by running HHblits [59] against nr90 sequence database (parameter setting: j: 3, id: 90, cov: 70) and constructed multiple sequence alignment of these proteins. **b** The correlation among residues in MSA was disentangled using composite likelihood maximization technique, generating prediction of inter-residue contacts. **c** The predicted contacts were fed into a deep neural network for refinement. **d** The refined prediction of inter-residue contacts
