# Supplementary material for: Predicting protein inter-residue contacts using composite likelihood maximization and deep learning
Source: BMC Bioinformatics. 2019 Oct 29;20:537. doi: 10.1186/s12859-019-3051-7 (PMC6821021; doi:10.1186/s12859-019-3051-7)
Supplement: Supplementary file 1 — The additional results on the performance of clmDCA. Figure S1 shows a case study of clmDCA. Figure S2 shows the comparison of the quality of the structures built using predicted contacts. Figure S3 shows the run time of clmDCA. Table S1 shows the time complexity for calculating likelihood function. Table S2 shows the performance of 3-order clmDCA. (PDF 721 kb) [file 12859_2019_3051_MOESM1_ESM.pdf]

Supplemental Materials: Predicting protein inter-residue contacts using composite likelihood maximization and deep learning

1. Calculation of gradients of composite likelihood

To find parameters h_i and e_{ij} such that $\mathcal{CL}_{\text{pairwise}}$ is maximized, we employed the classical BFGS technique with efficient calculation of gradients. The gradients of $\mathcal{CL}_{\text{pairwise}}$ can be calculated in polynomial time through the following equations.

$$\frac{\partial \mathcal{CL}_{\text{pairwise}}}{\partial h_i(a)} = (L-1)f_i(a) - \frac{1}{M}\sum_{m=1}^{M}\sum_{\substack{k=1\\k\neq i}}^{L} P_{i\hat{k}}^{m}(a) \quad (1)$$

$$\begin{aligned}\frac{\partial \mathcal{CL}_{\text{pairwise}}}{\partial e_{ij}(a,b)} &= (2L-3)f_{ij}(a,b) - \frac{1}{M}\sum_{m=1}^{M}[P_{ij}^{m}(a,b) \\ &+ \sum_{\substack{k=1\\k\neq i,k\neq j}}^{L}(I(X_j^m=b)P_{i\hat{k}}^{m}(a) + I(X_i^m=a)P_{\hat{k}j}^{n}(b))]\end{aligned} \quad (2)$$

Composite likelihood maximization is a tradeoff between model capacity and model complexity. Table 1 shows the time complexity of the calculation of the gradients of actual likelihood, pseudo-likelihood, and pairwise composite likelihood. Here, q denotes the alphabet size of amino acids types, L denotes the length of protein and M denotes the number of sequeces in MSA.

Likelihood function	Time complexity
Actual likelihood $\mathcal{L}$	NP-hard
Pseudo-likelihood $\mathcal{PL}$	$O(qL^2M)$
Pairwise composite likelihood $\mathcal{CL}$	$O(q^2L^2M)$

Table 1: Time complexity for calculating likelihood function.

2. Pre- and post-processing, regularization, and speed-up strategies

It is very common that some homologous proteins of a query protein show considerable sequence similarity, forming redundancy in MSA. This redundancy makes the assumption of independence among homologous proteins false and the likelihood function inaccurate. To reduce the impact caused by redundancy, we pre-processed MSA by weighting all homologous proteins within it as performed by plmDCA [2] and PSICOV [3]. Similar to [1], we also applied the average-product correction (APC) technique on the predicted contacts as post-processing procedure.

The MRF model used in this study contains a total of $O(L^2)$ parameters, usually exceeding the number of homology proteins in MSA. For example, about 2×10^7 parameters are needed to model a query protein with length $L = 300$;

in contrast, few MSAs have sufficient homologous proteins for estimating these parameters, thus causing potential overfitting. To avoid overfitting, we added a penalty term to the likelihood function as follows:

$$R_{l_2} = \lambda_h \sum_{i=1}^{L} \|h_r\|_2^2 + \sum_{i=1}^{L} \sum_{j=i+1}^{L} \|e_{ij}\|_2^2 \tag{3}$$

To speed up likelihood maximization, we began with initial parameters calculated using plmDCA. We also exploited the parallelism in calculating gradient for residue pairs and implemented our method using OpenMP. Overall, it usually takes only a few hours for clmDCA to predict contacts for proteins with typical length of 200 amino acids.

3. Comparison of plmDCA and clmDCA: a case study

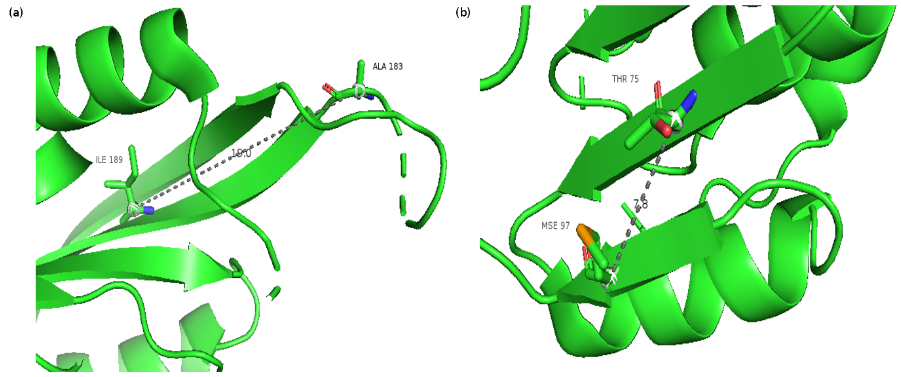

Figure 1: Two residue pairs in protein `1ne2A`. (a) Non-contact residue pair `ALA183-ILE189` and (b) Contacting residue pair `THR75-MSE97`.

4. Building protein 3D structures using the predicted inter-residue contacts

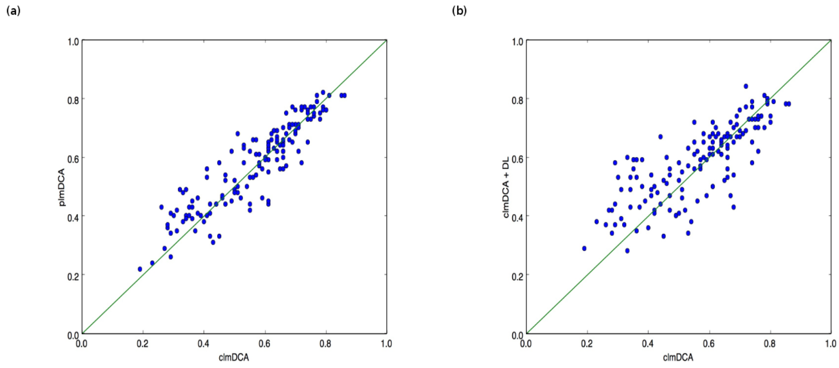

Figure 2: Comparison of quality of structures built using predicted contacts by (a) clmDCA vs. plmDCA. (b) clmDCA alone version clmDCA together with refinement using deep learning. Here protein quality is measured using TMscore. Data set: PSICOV.

5. Comparison of runtime

The runtime of clmDCA is mainly determined by the number of sequences of MSA and the columns of MSA. For runtime benchmarks, we generated synthetic MSAs with 3000 sequences and 50, 250 and 500 columns. 4 CPU cores are used for clmDCA, plmDCA and PSICOV. As shown in Figure 3, clmDCA is generally $1.5 - 2$ times slower than plmDCA. We will explore GPU techniques to further speed up clmDCA in our future work.

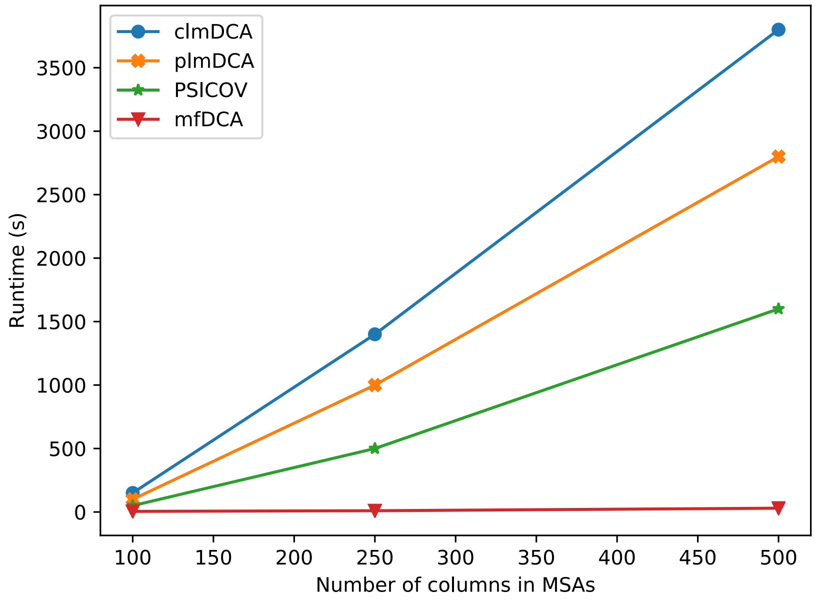

Figure 3: Comparison of runtime

6. Performance of 3-order clmDCA

Table 2: Contact prediction accuracy on CASP-11 targets.

Methods	*separation* ≥ 6				*separation* > 23			
	$L/10$	$L/5$	$L/2$	L	$L/10$	$L/5$	$L/2$	L
clmDCA	0.57	0.53	0.44	0.36	0.53	0.49	0.38	0.29
3-order clmDCA	0.56	0.53	0.46	0.36	0.54	0.49	0.40	0.28

References

[1] Dunn, S. D., Wahl, L. M. and Gloor, G. B. (2008) Mutual information without the influence of phylogeny or entropy dramatically improves residue contact prediction. *Bioinformatics*, **24**, 333–340.

[2] Ekeberg, M., Lövkvist, C., Lan, Y., Weigt, M. and Aurell, E. (2013) Improved contact prediction in proteins: using pseudolikelihoods to infer potts models. *Physical review. E, Statistical, nonlinear, and soft matter physics*, **87**, 12707.

[3] Jones, D. T., Buchan, D. W., Cozzetto, D. and Pontil, M. (2012) PSICOV: precise structural contact prediction using sparse inverse covariance estimation on large multiple sequence alignments. *Bioinformatics*, **28**, 184–190.
